# Supplementary material for: Ensemble learning based transmission line fault classification using phasor measurement unit (PMU) data with explainable AI (XAI)
Source: PLoS One. 2024 Feb 12;19(2):e0295144. doi: 10.1371/journal.pone.0295144 (PMC10861062; doi:10.1371/journal.pone.0295144)
Supplement: Attachment — Submitted filename: Reviewers_answers.docx [file pone.0295144.s001.docx]

Reviewer #1: Dear authors, I have read your paper, titled " Ensemble learning based transmission line fault classification using phasor measurement unit (PMU) data with explainable AI (XAI) ", carefully. I am of the convection that the paper needs some serious revisions before I recommend it for publication in this journal. Please necessarily apply the comments and highlight them in the paper.

The following presents my comments.

1. The Abstract section of the paper has been written very poorly. You are expected to state the necessity of your research and its novelty correctly. Please describe the software implementation and algorithm results briefly. I think the Abstract section needs to be 200-300 words and formatted in a standard way. Please revise the Abstract.

Thank you for bringing up this fact. We appreciate your acknowledgment. We have revised our **Abstract** section and this time we have illustrated the necessity and novelty of our research more specifically. Additionally, the data generation process, software implementation, and the outcomes from our approach have been discussed briefly in the **Abstract** section.

2. The Introduction section has been written acceptably but it is recommended to divide it into five parts as follows: (a)Research motivation, (b) literature review, (c) the necessity of the research based on challenges of the literature, (d) novelty and main contributions of the paper, and (e) organization and structure of the paper.

We appreciate your acknowledgment. We have divided the **Introduction** section into five parts sequentially including **Research motivation**, **Literature review**, **Necessity of the research**, **Novelty and major contributions**, and **Organization and structure** according to the given recommendation and described each subsection with appropriate arguments.

3. The output results of the simulation are very brief. In this section, it is better to present more graphical and numerical results in more scenarios that can show the accuracy of the proposed algorithm well. In this section, you should be able to defend your algorithm by providing appropriate simulation results.

We appreciate your acknowledgment. We have added a new table, **Table 8** in **Experiment and Result Analysis** section to compare our fault classification technique with the existing approaches. This comparison supports the effectiveness of our model to classify faults as our model have been performed better than the outcomes achieved from recent studies in similar fields. However, in the **Experiment and Result Analysis** section, we have represented the analysis of the results thoroughly:

i. In **Table 5**, train and test accuracy for all the tested models have been represented. We have conducted a comparison of all the tested models based on their achieved accuracy scores.

ii. To check if the model is overfitted or not, we have presented cross-validation scores for best-performing models in **Table 6.**

iii. In **Fig 17**, To illustrate the performance in terms of each class, confusion matrices for the best-performing models have been presented that gives a comparison between actual and predicted values.

iv. Classification reports have been presented in **Table 6** which provides precision, recall, and f1-score for all the tested models. It ensures the evaluation of the models more preciously.

v. As we have performed ensemble learning and combined the predicted performances of best-performing models through soft voting, the voting report to show the contribution of each model to increase the predicted performance of the final model has been visualized in **Fig 18**.

vi. Receiver operating characteristic (ROC) curve for multiclass has been presented in **Fig 19** for the ensemble model to evaluate the performance of the ensemble model against each class.

We have evaluated the developed model with different performance matrices to support the effectiveness of our developed model in **Experiment and Result Analysis** section. We have compared different classifier outcomes to identify the best-performing models. Performance against each class has been shown for the individual best-performing models and also for the combined model accordingly.

4. Sensitivity of the proposed algorithm should be analyzed with respect to fundamental parameters. Please add a new item title named "Sensitivity Analysis" and test the sensitivity of the algorithm to different parameters and conditions and provide the results in numerical and graphical forms.

Thank you for addressing this question. With the aim of enhancing comprehensibility for readers, we have added a new section **Sensitivity Analysis with Table 5 and Fig 17** to our research. We divided the dataset into multiple subgroups and then used these subgroups both individually and combined with other subgroups as the input to the train ensemble model. Finally, we have compared the obtained outcomes to check the sensitivity of the developed model in terms of different parameters.

Reviewer #2: Following are the comments for improvement:
1. Why are the researchers considering values of reactive power in milli i.e., mVA, power in mW?

Thank you for bringing up this particular matter. We deeply regret to inform you that an error occurred during the writing process, resulting in a misinterpretation of the labeling format for reactive power. We sincerely apologize for any confusion or inconvenience this may have caused. We have rectified this error and revised the labeling in the **Data Generation** section. Specifically, we have replaced the previous labeling with the appropriate units of MVA (mega volt-ampere) and MW (megawatt) in order to accurately represent the reactive power measurements.

2. The results are very blurred. It must be improved before taking into consideration for further review.

Thank you for your effective identification. All the figures have regenerated and this time figures' dots per inch (DPI) have been set to 300 also quality has increased through AI tools for specific pictures including **Figure 11**, **Figure 13**, **Figure 14**, **Figure 15**, **Figure 16**, **Figure 20**, and **Figure 20**. The instructions specifying the areas that require improvement, will be more effective for us to make changes.

3. Mathematical model must be added for transmission line under different fault conditions.

We appreciate your acknowledgment. However, the mathematical model will be inappropriate for our research as we proposed a completely different approach to classify transmission line faults. The mathematical model is an equivalent circuit representation of a transmission line, consisting of series impedance (Z), shunt admittance (Y), and input/output nodes. It helps analyze transmission line behavior under different conditions by manipulating the circuit. On the other hand, our approach is an artificial intelligence (AI) based model that can classify different faults by identifying a pattern through analyzing the historical data of different fault conditions. We have trained different machine learning multiclass classifiers using the historical faulty labeled data of transmission lines and identified different transmission line faults based on the given parameters. To sum up, it can be said these two approaches can be considered as substitutes for one another. Hence, the implementation of the mathematical model for each fault type won’t be appropriate for our research.

4. Ensemble learning is used by authors? Any benefits over advanced learning methods already available in literature.

We appreciate your acknowledgment. However, we have represented how our fault classification approach has performed better than the existing approaches in similar fields in the **Experiment and Result Analysis (Table 8)** section. We have retrieved a multivariate dataset to classify faults and identified nine features as the ideal input after pre-processing the dataset. Our intention is to extract features from phasor measurement (PMU) data through exploratory data analysis (EDA) as we can show the effectiveness of the phasor measurement unit (PMU) data to classify transmission line faults, which is a vital factor of our research. Hence, we used machine learning techniques to address this issue, which has enabled us to extract features through data analysis not with the help of convolution layer piles and sets of pooling layers. Our approach ensures more interpretability and explainability compared to other approaches by providing more transparent and interpretable models, enabling a better understanding of how the input features influence the output. In light of this, we can say that our approach requires explicit feature engineering, where domain knowledge is utilized to extract relevant features from the data. This can be advantageous when there is prior knowledge about the problem domain and specific features that are meaningful for the task at hand. Additionally, the dataset that we have generated performed reasonably well in the machine learning algorithms rather than other approaches. The three machine learning models outperformed to predict transmission line faults, as well as these models, performed considerably well in IEEE 14 bus systems since we have made the decision to combine the performance of these models through soft voting based on knowledge gathered from the literature review, which represented how the effect of simulation tests in this field reflect in real life. Literature review related to this field has shown evaluation through simulation is considerably reliable so we can rely that the combined performance of the three models can handle real-life scenarios as well.

5. Testing, training and validation is not explained.

We appreciate your acknowledgment. However, in the **Data Preparation** section, we have explained the details of the train and test set which includes the train-test splitting, count of data in the train-test set, and label encoding of the target feature. In short, the workflow of data preparation from separating the inputs and target to feeding it to the machine learning models has been visualized in the **Data Preparation** section. In **Table 5** of the **Experiment and Result Analysis** section, both train and test accuracy for all the tested models has been interpreted. Additionally, in **Table 6** of the **Experiment and Result Analysis** section, the cross-validation score has been interpreted for best-performing models.

6. 10 fold validations? Why any specific reason?

Thank you for addressing this question. With the aim of enhancing comprehensibility for users, we have added the reason for using 10-fold in the **Methodology** section. The 10-fold cross-validation technique has been employed in this study to ensure an ample amount of data is available for training and validation purposes. This method divides the dataset into ten equal-sized subsets or "folds." During each fold, 90% of the data is utilized for training the model, while the remaining 10% is allocated for validation. This division allows for a substantial training dataset, which enhances the model's ability to learn effectively. Moreover, the validation set in each fold is sufficiently large, enabling a reliable estimation of the model's performance. By utilizing 10-fold cross-validation, we have achieved a more stable assessment of the model's performance compared to using a smaller number of folds. Similarly, the utilization of an increased number of folds in the cross-validation process has resulted in a notable reduction in the sensitivity of the estimated performance to the particular partitioning of the data.

7. Data preprocessing " It is noticeable that in terms of no fault cases value of current and voltage phases are normally distributed." How? and Why? It can be some other distribution as well.

We appreciate your acknowledgment regarding the reference made to this matter. We have conducted the distribution check to identify outliers in the data. As in no-fault conditions, data (current & voltage) are symmetrically distributed with no unexpected skew. Additionally, we have calculated skew and kurtosis values for the no-fault condition, which is in the -2 to 2 and -7 to 7 range respectively. These findings indicate that the data are normally distributed during the no-fault condition. We have discussed these facts in the **Preprocessing** section.

8. The information related to outliers are not explained.

Thank you for addressing this question. We appreciate your acknowledgment regarding the reference made to this matter. We have added a discussion of the technique that is used to handle the outliers in the **Preprocessing** section. We have used the interquartile range (IQR) technique that removes the data points outside the range of 1.5 times. Any observations that have been found more than 1.5 IQR below Q1 or more than 1.5 IQR above Q3 have been considered outliers and removed from the dataset.

9. The limitations of the research work must be added.

Thank you for addressing this question. We have mentioned the limitation of this research in the **Conclusion** section. As for now, the physical setup to test this model hasn't been ready as well as real-time labeled data of the transmission lines is also limited, so our developed model has only been tested on simulation-based platforms. This boundary has been considered the limitation of this research.

10. RF, KNN, Decision Tree, Ensemble results are not fully added. Some of the results are missing for the mentioned techniques.

We appreciate your acknowledgment. We have added a new section **Sensitivity Analysis** with **Table 5** and **Fig 17** to demonstrate the obtained outcomes from similar groups of features. In this section, we have used separate groups of parameters individually and also combined them one by one with other groups as the input of the developed model to analyze the sensitivity of the ensemble model in terms of different parameters. This section represents the model outcomes obtained by the developed ensemble model more enormously. However, in the **Experiment and Result Analysis** section, we have thoroughly represented the analysis of the results obtained from Decision Tree, RF, KNN, and Ensemble learning:

i. In **Table 5**, train and test accuracy for all the tested models have been represented. We have conducted a comparison of all the tested models based on their achieved accuracy scores and identified DT, RF, KNN, and the Ensemble model as the best-performing model.

ii. To check if these selective models (DT, RF, KNN, and Ensemble) are overfitted or not, we have presented cross-validation scores across 10 fold in **Table 6.**

iii. In **Fig 17**, To illustrate the performance in terms of each class, confusion matrices for the best-performing models (DT, RF, KNN, and Ensemble) have been presented that give a comparison between actual and predicted values.

iv. Classification reports have been presented in **Table 6** which provides precision, recall, and f1-score for all the tested models including DT, RF, KNN, and Ensemble. It ensures the evaluation of the models more preciously.

v. As we have performed ensemble learning and combined the predicted performances of best-performing models through soft voting, the voting report to show the contribution of each model to increase the predicted performance of the final model has been visualized in **Fig 18**.

vi. Receiver operating characteristic (ROC) curve for multiclass has been presented in **Fig 19** for the ensemble model to evaluate the performance of the ensemble model against each class.

We have evaluated these selective (DT, RF, KNN, and Ensemble) models with different performance matrices to support the effectiveness of our developed models in the **Experiment and Result Analysis** section. We have compared different classifier outcomes to identify the best-performing models. Performance against each class has been shown for the individual best-performing models (DT, RF, and KNN) and also for the combined model (Ensemble Model) accordingly.

11. Correlation matrix ? Whta is the significance of it?

We appreciate your acknowledgment regarding the reference made to this matter. We have discussed the significance of the correlation matrix in the **Feature Extraction** section. We have identified 12 features from the phasor measurement unit (PMU). However, we have considered only 9 features as the input since there exist correlations among certain input features, which has a significant possibility to generate biased and wrong predictions for the model. Hence, we checked the impact of each feature and keep the most impactful features from the correlated features. Additionally, we have added a new section **Sensitivity Analysis (Fig 17)** in our research, which cross-verified the fact that correlated features have affected the predicting performance of the developed model.

12. Region of Occurrence ? Why? Analysis must be done with supported technical arguments.

Thank you for addressing this question. We appreciate your acknowledgment regarding the reference made to this matter. We have added a subsection **0.5 Organization and structure** under the **Introduction** section that demonstrates where we can implement the proposed model and how it can affect the system. The primary goal of this research is to develop an artificial intelligence platform that utilizes Fourth Industrial Revolution (4IR) technologies for the purpose of monitoring the power grid. The proposed model aims to possess the ability to accurately identify and categorize short circuit events in various real-world scenarios. To achieve this, it is intended to deploy the developed model at different locations along the physical transmission line. This deployment strategy will enable the acquisition of comprehensive information regarding any instances of blackouts or other anomalous events that may transpire within the electrical grid.

13. Comparative table must be added in order to support the proposed method as compared to existing methods.

Thank you for addressing this question. We appreciate your acknowledgment regarding the reference made to this matter. We have added a new table, **Table 8** in **Experiment and Result Analysis** section to compare our fault classification technique with the existing approaches. This comparison supports the effectiveness of our model to classify faults as our model have been performed better than the outcomes achieved from the recent studies in similar fields.

14. IEEE 14 bus system is mentioned in abstract. No details are added in the research about it.

Thank you for addressing this question. We have added a demonstration of the evaluation process through the IEEE 14-bus system at the end of the **Experiment and Result Analysis** section. As IEEE 14 bus is a popular IEEE benchmark bus system so we didn’t explain the components of the bus system. However, we discussed how we have used the 14-bus to evaluate our model. We have exported our ensemble model within the IEEE 14 bus system in .mat format to assess its capabilities. In order to evaluate its predictive accuracy, we generated various fault scenarios using fault circuits and utilized our developed model to predict these scenarios. Additionally, we conducted individual tests of the best-performing models on the 14-bus system to determine their individual effectiveness.

Reviewer #3: The paper presents an ensemble learning based Transmission line fault detection and classification. Following are my comments.

1. The citation of the references order in the manuscript is random i.e. The first citation begins with a reference[19]. It should be serially in incremental order.

Thank you for addressing this question. We appreciate your acknowledgment regarding the reference made to this matter. We have rearranged the references in incremental order. We sincerely apologize for any confusion or inconvenience this may have caused.

2. Authors are claiming the cutting-edge accuracy at 99.88% on Synthesized data, what is the guarantee that the sysnthesized result will reflect in real-time in physical transmission line.

We appreciate your acknowledgment regarding the reference made to this matter. To validate the performance of our model, conducting physical experiments in a controlled environment is currently unfeasible due to the extensive equipment setup required. As an alternative, we have deployed our ensemble model within the IEEE 14 bus system to assess its capabilities. In order to evaluate its predictive accuracy, we generated various fault scenarios using fault circuits and utilized our developed model to predict these scenarios. Additionally, we conducted individual tests of the best-performing models on the 14-bus system to determine their individual effectiveness. It is worth noting that within the realm of research in this field, evaluating AI models in real-world settings poses significant challenges. Consequently, many studies outline the evaluation of their developed models using IEEE benchmark bus systems. We have demonstrated the evaluation process through the 14-bus system in the **Experiment and Result Analysis** section.

3. It is suggested to the authors to perform the same analysis based on the data from the physical transmission line or practical laboratory setup, and compare the accuracy with simulated results.

We appreciate your acknowledgment. Unfortunately, due to the unavailability of labeled real-time data pertaining to transmission line fault cases, we are unable to conduct testing of our model using data from a physical transmission line or a practical laboratory setup. However, we have rigorously evaluated and validated the performance of our model through simulation scenarios. We have demonstrated the evaluation process through the simulation in the **Experiment and Result Analysis** section. This approach holds a consistent validation method utilized in numerous studies within this field. Nevertheless, we have added this fact as the limitation of this research in the **Conclusion** section.

4. In the real-time transmission line the voltage, current and the power in higher scale. The simulated results are performed in Milli volt and Milli watt level, What is the practicality of applying the proposed methodology in physical real-time transmission line.

We appreciate your acknowledgment regarding the reference made to this matter. During the writing process, there was a misinterpretation of the labeling format for reactive power. We sincerely apologize for any confusion or inconvenience this may have caused. We have rectified this error and revised the labeling in the **Data Generation** section. Specifically, we have replaced the previous labeling with the appropriate units of MVA (mega volt-ampere) and MW (megawatt) in order to accurately represent the reactive power measurements.

5. The resolution of some figures is very poor (such as Fig.6, Fig.7, Fig.11, Fig.14) it need to be improved.

Thank you for your effective identification. All the figures have regenerated and this time figures' dots per inch (DPI) have been set to 300 also quality has increased through AI tools for specific pictures including **Figure 11**, **Figure 13**, **Figure 14**, **Figure 15**, **Figure 16**, **Figure 20**, and **Figure 20**.

6. PLOS authors have the option to publish the peer review history of their article ([what does this mean?](https://journals.plos.org/plosone/s/editorial-and-peer-review-process#loc-peer-review-history)). If published, this will include your full peer review and any attached files.


If you choose “no”, your identity will remain anonymous but your review may still be made public.

No, I want my identity to remain anonymous.
