# Supplementary material for: Ensemble learning based transmission line fault classification using phasor measurement unit (PMU) data with explainable AI (XAI)
Source: PLoS One. 2024 Feb 12;19(2):e0295144. doi: 10.1371/journal.pone.0295144 (PMC10861062; doi:10.1371/journal.pone.0295144)
Supplement: Attachment — Submitted filename: Reviewer_answer_2nd.docx [file pone.0295144.s002.docx]

Reviewer #2: The manuscript is improved. However, following issues must be addressed.

1. However, authors must address the time required for classification of faults.

Thank you for addressing this matter promptly. I have added this information in the **Experiment and result analysis** section under the description of **Fig 20**. 26878 test samples, belonging to six distinct classes, were classified as clean and faulty instances in 0.4977 seconds by the proposed hybrid model.

2. The figures still require improvements.

Thank you for your kind consideration of this issue. The overall figure quality has seen significant improvement, with particular attention given to enhancing the clarity and resolution of images considering “figure text font size must match the paper body font size considerably”. All figures have been incorporated into the paper and their quality has been appropriately adjusted to meet the standards this time. While certain libraries were employed to gain deeper insights into the study through data analysis and explainable AI techniques, it was noted that the initial image outputs from these libraries did not meet the desired level of quality. However, through the application of advanced AI tools, the image quality has been successfully elevated to its highest standard. It is worth highlighting that meticulous care was taken to ensure that all generated images adhere to the stipulated standard, with a uniform DPI setting of 300 across the board.

3. An analysis should also be added to verify that the PMUs with this approach work exactly the same for distribution line and transmission lines so that this could be applicable to any power lines.

I appreciate your prompt attention to this matter, as it enhances the comprehensiveness of the research. In distribution systems, PMUs are often referred to as "Micro PMUs" or "µPMUs". These devices are designed for use in low-voltage distribution networks, which are the parts of the grid that deliver electricity directly to end-users. However, in transmission systems, standard PMUs are commonly used, and they are not typically referred to as "Micro PMUs". These devices are designed for use in high-voltage transmission networks, which transport electricity over long distances. Distribution line PMUs are typically deployed at lower voltage levels and are used for monitoring and control of distribution system parameters, such as voltage, current, and power quality. Transmission line PMUs are used for monitoring and controlling parameters in high-voltage transmission lines, such as phase angles, frequency, voltage magnitudes, and line impedance. The PMUs used in distribution networks (Micro PMUs) and transmission networks (standard PMUs) are fundamentally different devices designed for different voltage levels and purposes, and their data parameters are not the same. Therefore, we are not claiming that the approach works exactly the same for distribution lines and transmission lines. However, we think this can be a good future direction based on our current research on transmission line fault classification using ML approaches.

4. The effect of synchronous condenser seems neglected on fault classification in IEEE 14 bus system.

I sincerely appreciate your attention to this matter, as it contributes to a more comprehensive examination. In Transmission Line Short Circuit Fault Analysis using Machine Learning, the synchronous condenser or synchronous motor effect is not a primary focus. Typically, fault analysis in transmission lines focuses on identifying and classifying different types of faults, such as short circuits or line-to-ground faults, based on frequencies, phases, voltage, and current measurements. While the synchronous condenser or motor effect does influence the transient behavior of the power system during faults, it is generally a well-understood and predictable phenomenon. Accordingly, the complexity introduced by modeling the synchronous condenser or motor effect does not provide significant benefits for the specific task of fault analysis using machine learning. Machine learning models are designed to learn patterns and anomalies in the data to identify faults accurately. Hence, adding the complexity of modeling synchronous machines does not substantially improve the model's performance for fault detection and classification. These models primarily rely on provided measures including frequency, phase angle, voltage, and current data without delving deeply into synchronous machine dynamics. It has been observed the synchronous condenser or synchronous motor effect is an important consideration in power system analysis and design, but it is not a primary focus in the specific context of machine learning-based Transmission Line Short Circuit Fault Analysis.
