# Supplementary material for: Ensemble learning based transmission line fault classification using phasor measurement unit (PMU) data with explainable AI (XAI)
Source: PLoS One. 2024 Feb 12;19(2):e0295144. doi: 10.1371/journal.pone.0295144 (PMC10861062; doi:10.1371/journal.pone.0295144)
Supplement: Attachment — Submitted filename: Response to Reviewers.docx [file pone.0295144.s003.docx]

Hafiz Abdur Rahman, Ph.D.

Professor, North South University

8 November, 2023

Praveen Kumar Donta, Ph.D.

Academic Editor

PLOS ONE

Dear Dr. Kumar Donta,

Re: PONE-D-23-11739R2

Title: Ensemble learning based transmission line fault classification using phasor measurement unit (PMU) data with explainable AI (XAI)

I would like to express my sincere gratitude for taking the time to review our manuscript and for providing valuable feedback. We appreciate the constructive comments and suggestions made by the editor and reviewers.

**Journal Requirements:**

**Please review your reference list to ensure that it is complete and correct. If you have cited papers that have been retracted, please include the rationale for doing so in the manuscript text, or remove these references and replace them with relevant current references. Any changes to the reference list should be mentioned in the rebuttal letter that accompanies your revised manuscript. If you need to cite a retracted article, indicate the article’s retracted status in the References list and also include a citation and full reference for the retraction notice.**

The reference list has undergone a thorough review and the DOI (Digital Object Identifier) for references numbered 46, 52, and 53 has been revised and updated. Within the literature review section, there has been an update to reference number 31 in the summary table. Additionally, a new reference has replaced the content previously associated with reference number 59.

**Reviewers' comments:**

**Reviewer #2: The manuscript is much improved. The protection time required for different type of faults must be tabulated for extensive understanding.**

I appreciate the reviewer's feedback on incorporating protection time requirements based on the control domain of power systems for different types of transmission line faults. While this is indeed a valuable aspect of power system operation and protection, it is essential to clarify that the primary objective of our research is to develop a fault classification framework within the monitoring domain. As such, my work focuses on the accurate identification and categorization of transmission line faults, which can significantly aid in the early detection and diagnosis of faults, supporting overall system reliability and maintenance.

Transmission line fault classification using machine learning (ML) and the determination of protection time requirements and relay-based control are distinct components within power system analysis. Fault classification with ML aims to enhance early fault detection and diagnosis through data-driven models, primarily serving the monitoring domain, and facilitating improved situational awareness. In contrast, protection time requirements and relay-based control, situated in the control domain, focus on designing protective relays and schemes to ensure swift and selective fault isolation, reducing system disturbance duration and safeguarding equipment. These two areas address separate aspects of power system engineering, with fault classification primarily enhancing monitoring capabilities, while protection time requirements and relay-based control are pivotal for maintaining grid reliability and minimizing damage.

As refference: <https://doi.org/10.1016/j.engappai.2021.104504>, <https://doi.org/10.1063/5.0108588>. These articles illustrate the significant role of machine learning in the identification of faults within power systems.

The implementation of protection time requirements and relay-based control is undoubtedly an important topic in the power domain, but I intend to consider it as a potential avenue for future research, as it falls outside the scope of the current study.

Sincerely,

Hafiz Abdur Rahman, Ph.D.

hafiz.rahman@northsouth.edu
